# A Mobile Robots Experimental Environment with Event-Based Wireless Communication

**DOI:** 10.3390/s130709396

**Published:** 2013-07-22

**Authors:** María Guinaldo, Ernesto Fábregas, Gonzalo Farias, Sebastián Dormido-Canto, Dictino Chaos, José Sánchez, Sebastián Dormido

**Affiliations:** 1 Department of Computer Science and Automatic Control, UNED, Juan del Rosal 16, Madrid 28040, Spain; E-Mails: efabregas@bec.uned.es (E.F.); sebas@dia.uned.es (S.D-C.); dchaos@dia.uned.es (D.C.); jsanchez@dia.uned.es (J.S.); sdormido@dia.uned.es (S.D.); 2 School of Electrical Engineering, Pontificia Universidad Católica de Valparaiso, Av. Brasil 2147, Valparaiso, Chile; E-Mail: gonzalo.farias@ucv.cl

**Keywords:** distributed control systems, mobile robots, multi-agent systems, wireless communications, embedded systems

## Abstract

An experimental platform to communicate between a set of mobile robots through a wireless network has been developed. The mobile robots get their position through a camera which performs as sensor. The video images are processed in a PC and a Waspmote card sends the corresponding position to each robot using the ZigBee standard. A distributed control algorithm based on event-triggered communications has been designed and implemented to bring the robots into the desired formation. Each robot communicates to its neighbors only at event times. Furthermore, a simulation tool has been developed to design and perform experiments with the system. An example of usage is presented.

## 1. Introduction

Significant developments in the fields of communication technology, wireless technology, embedded devices, and many others, have enabled the development of autonomous air, ground, or underwater vehicles. Groups of such vehicles, referred to as agents, can be utilized to solve a variety of problems very efficiently, such as for example exploration and monitoring tasks.

One of the main problems in these systems is when the control task is centralized. This centralization demands a very powerful communication network to send state information in a timely manner and detailed models of the mobile robot interactions. Both of these requirements can greatly limit the scalability of centralized approaches in networked control systems. An alternative way is to use a distributed approach [1,2], where each mobile robot decides its actions based only on his own state and the direct neighbors' information, while guaranteeing that the whole group fulfills a common group objective. One example of a group objective for multi-agent systems is state agreement or consensus, *i.e.*, all agents are supposed to converge to a common point or state. Such consensus problems have a variety of applications in flocking, attitude synchronization in satellite swarms, distributed sensor networks, congestion control in communication networks, or formation control [3]. We are particularly interested in the last field of application since achieving a stable formation is analogous to reaching consensus. In this consensus problem the agents communicate through a network exchanging information to reach the desired formation. Specifically, we propose to incorporate wireless links between the mobile robots to support the communications, the embedded systems, and the control.

Moreover, all real networks have bandwidth limitations that can cause delays in message delivery producing a major impact on overall system stability [4]. In this sense, it is desirable to know how frequently mobile robots should communicate such that the system maintains a desired level of performance. In this light, event-based communications are an alternative to time-scheduled control [5], and they have been in the focus of many researchers in recent years such as [6–11], to mention just a few. In the literature, several simplifications are usually done in both the robot dynamics and the network models of a multi-agent robot system. However, a real robot has its own physical parts, including sensors, processors, actuators, and communication devices. Therefore, its limitations must be considered, such as those related to computational power, sensor errors, and actuating and communicating devices.

This paper presents a multi-agent approach through the use of a decentralized control with event-triggered communication in a mobile robot formation control with a consensus algorithm. The decentralized control is less vulnerable to a node failure and easier to scale than the centralized approaches presented in [12–14]. In [12] the formation control of a multi-Khepera robot system is solved without communication between robots. However, each robot executes an algorithm periodically that requires the knowledge of the complete graph of the formation, so it is computationally intensive for large number of robots. Centralized approaches are proposed in [13,14]. In [13] all complexity of sensing, control, and computation is relegated to the leader of a group of robots, and [13] treats the robots formation as a single virtual rigid structure.

A decentralized approach for cooperative control is presented in [15] and a detailed hardware description is given and several experimental examples of multi-vehicle control for TXT-1 monster trucks from Tamiya, Inc, are given for both communication and vision formation control. In [16] a hardware platform for a single vehicle is developed and a PID event-based control is implemented. However, none of these works consider distributed event-based control in a multi-robot environment. Hence, the main contribution of this paper is the implementation of distributed event-triggered algorithms in a real mobile robots platform since, to the best knowledge of the authors, all the results in this field are mainly theoretical and only present simulation results. The experimental results show the goodness of the event-trigger approach compared to time-driven control, reducing the communication between agents. Furthermore, in contrast to other works, as for example [12,15], in which a costly platform for indoor or outdoor experimentation is used, we give a detailed description of our low-cost hardware platform, and how it was implemented in LEGO robots, in order to help readers who are considering building a similar system for research, educational purposes [17], or to mimic real world exploratory applications [18].

An interactive simulation, which is available at http://lab.dia.uned.es/mass, is also presented as a complement to the experimental setup. This simulation helped in the design and trial of different experiments with variable system and network conditions.

The paper is organized as follows. Section 2 describes in detail the experimental environment and its components. Section 3 gives a background on event-based control and multi-agent systems, and the extension of these to the formation control of a multi-robot system. Section 4 describes the simulation tool and an example of usage. Section 5 discusses experimental results and compares them to simulation results and experiences based on time-driven control. Finally, Section 6 ends the paper with conclusions and suggestions of future works.

## 2. Test Platform Description

We next describe the experimental environment designed with low-cost components. This may help readers to build similar systems. It can also be scaled up or down to equip bigger or smaller robots, respectively [19,20].

### 2.1. Platform Architecture and Components

**Figure 1.**
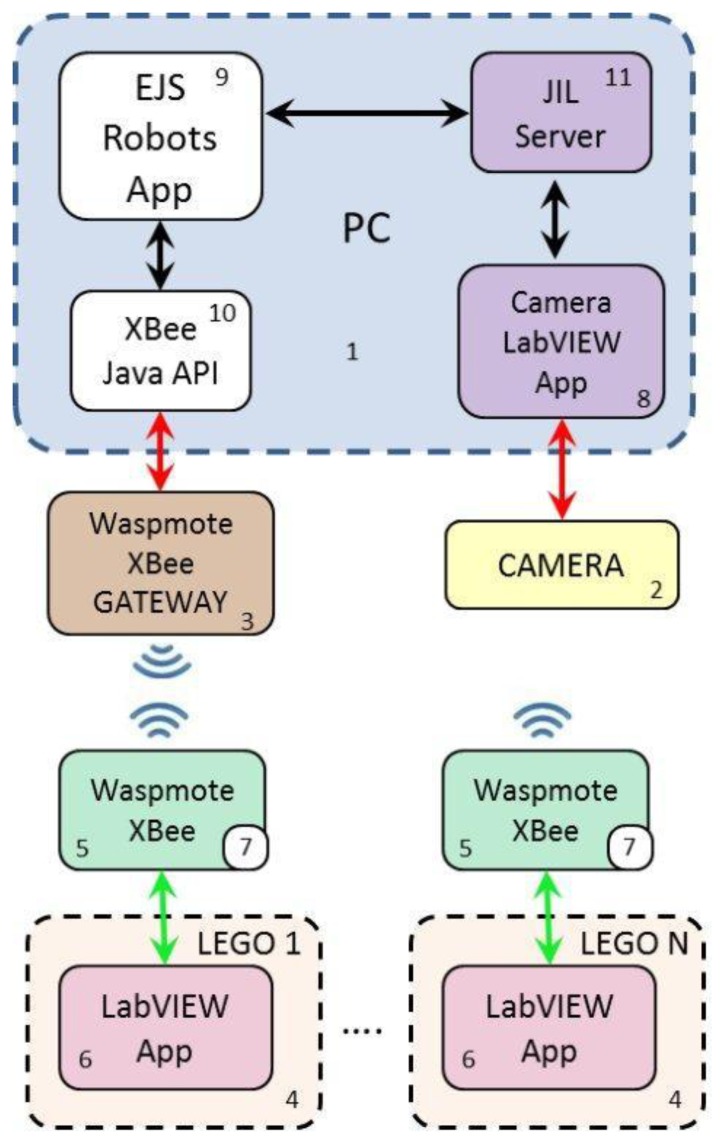
Block diagram of the platform architecture.

The developed tool is a test platform for multi-mobile robot systems. It consists of two main components: the vision and the communication systems. Different software tools exchange information to transform the images captured by the camera into positioning data, which are sent to the robots via wireless communications. Figure 1 shows the block diagram of the architecture and the components of the platform, which are the following:

1. A personal computer (PC) to calculate and send the position and orientation to the robots.
2. A CCD camera (charge-coupled device) connected to the PC and equipped with wide angle optics. The camera is placed in the ceiling of the room, as described in [21].
3. A Waspmote Gateway card connected to the PC [22].
4. A group of LEGO Mindstorms NXT robots with the HiTechnic IR RC Car configuration [23,24].
5. A Waspmote card connected to each NXT LEGO Mindstorms robot by I^2^C port [25].
6. A control and communication application, developed in LabVIEW, for each NXT LEGO robot.
7. An application, written in C++, for each Waspmote card.
8. An application (running on the PC), developed in LabVIEW, for the interaction with the camera [21].
9. A Java application, built with Easy Java Simulations (EJS) [26], for communication between the robots and the PC.
10. A Java XBee API to send and receive data through the Waspmote Gateway card [27].
11. The JIL server [28] to link LabVIEW with EJS.

### 2.2. Vision System

Though LEGO platforms provide an interesting group of sensors such as ultrasonic, touch, or light sensors, there is no device for measuring absolute positions. This makes experimentation in robot formation control difficult. One option to solve the previous drawback is the use of a GPS as the main localization sensor as in [29]. However, when the experiments are performed indoors, GPS is impractical.

**Figure 2.**
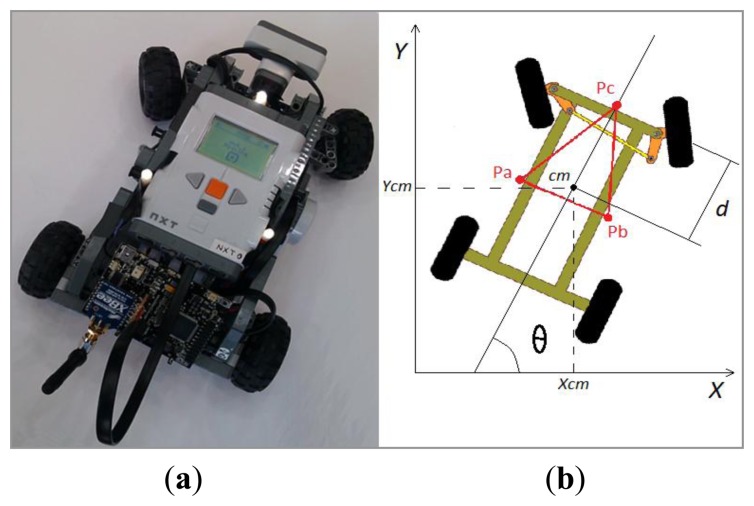
(**a**) The NXT LEGO robot, (**b**) Isosceles triangle formed by the LEDs.

Another alternative is to incorporate a vision system to get the robots poses. The recent appearance of RGB-D cameras has made possible 3D mapping from a single image and state estimation. However, running the robot real-time state estimation algorithms on board would require a powerful computer, which would increase the cost of the platform considerably. Thus, a single camera on the ceiling observes the arena from an overhead position. Each robot can be distinguished thanks to three high brightness LEDs. These light spots form an isosceles triangle with different aspect ratio for each robot as shown in Figure 2a. In Figure 2b, the points *Pa, Pb*, and *Pc* represent the LEDs. The orientation is denoted by *θ*. The position of each robot is calculated using their center of mass (*Xcm; Ycm*).

The Basler A631fc CCD camera (block 2 of Figure 1) is configured in monochrome mode for image acquisition in order to maximize the resolution and shutter speed. Under these conditions, we obtain images with a resolution of 1,388 × 1,038 pixels at 18.7 fps.

The images captured by the camera are processed by the LabVIEW Camera App (block 8 of Figure 1), which computes the robots poses. The execution of the application implies the following steps:

- *Barrel distortion correction:* The image magnification decreases with distance from the optical axis. The apparent effect means that an image has been mapped around a sphere (or barrel).
- *Perspective transformation:* Transforms the coordinates of the object to the camera coordinates, making rotation and translation transformations.
- *Calculation of positions and orientations:* From the reference points on the ground, we calculate the position and orientation taking into account the morphology of the obtained points.
- *Transformation of coordinates:* From pixels to meters.
- *Calibration:* Gets the perspective calibration parameters.

**Figure 3.**
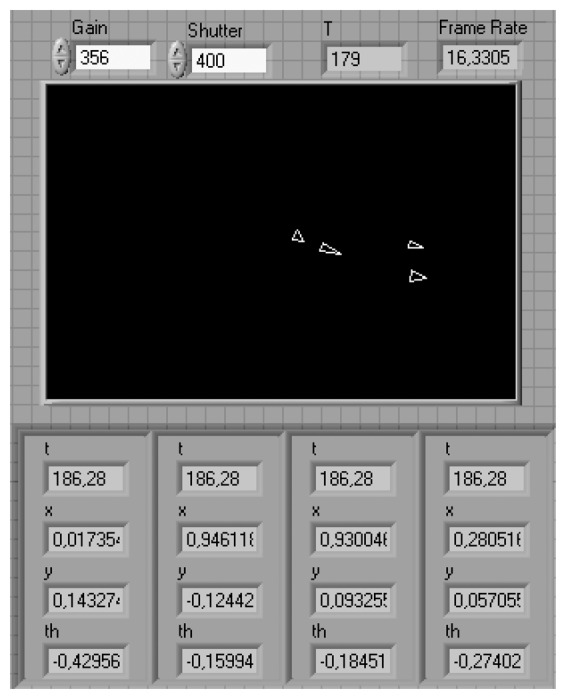
User interface of the Camera LabVIEW App.

The front panel of the LabVIEW Camera App is shown in Figure 3. The values of the configuration of the camera (gain, shutter, and frame rate) and the period (T) are required to compute the bearings, and shown on the front panel. The application sends the absolute position and orientation to the robots every 200 milliseconds. Further details about this application can be found in [21].

### 2.3. Communication System

After the processing of the robots poses, they are sent to the robots. Each one receives its position and orientation, and decides when to broadcast this information to its neighbors based on the local data, updating the control law afterwards. Thus, the control is distributed in the sense that each robot takes by itself the decision of when to transmit its state and the control law is computed locally, in contrast to [16], where the control law is computed on the central computer and sent to the robots.

Waspmote boards (block 5 of Figure 1) are used for the wireless communications. This card has a modular architecture and is based on the Atmel ATMEGA 1281 microcontroller. The Waspmote also integrates the Digi XBee RF (ZigBee standard) module for communication in the ISMB bands (Industrial Scientific Medical Band, RF-868 MHz) [30]. This module communicates with the microcontroller via interruptions using the UART at the speed of 38,400 bps. It allows the definition of different network configurations (e.g. p2p, tree, or mesh) and uses the protocol 802.15.4 with a frequency of 2.4 GHz [22]. For wireless network settings, each card uses the MAC address of its corresponding XBee. Each robot “talks” to its neighbors given by the communication graph (see Sections 3 and 4.2 for further details).

The power supply for the Waspmote is a lithium battery. The energy system has four operational modes (on, sleep, deep sleep, and hibernate). In normal operating mode (on), the consumption is 9 mA. Every 125 ns the microcontroller runs a routine of low level (machine language) instructions [31,32], which is fast enough to the time requirements of our system.

The I^2^C bus interfaces the NXT LEGO with the Waspmote. The NXT LEGO runs as master and the Waspmote as slave. The Waspmote card runs an application (block 7 Figure 1) that allows the LEGO NXT to communicate with other robots and the PC. Every 100 ms the following tasks are executed:

1. It checks if a packet has been received by the XBee radio. Packets can be sent from the PC or from another robot. If the answer is positive, the received data is stored in a buffer until the NXT LEGO requires it.
2. It checks if a flag is true. If the answer is positive, it indicates that the NXT LEGO requires the sending of a packet to other robots (its neighbors) by Xbee radio. Once the packet is sent the flag is set to false. The NXT LEGO triggers an interruption routine (which sets the flag to true) when a packet must be sent. In event-based control, the flag is set to true at the event occurrence time.

Each NXT LEGO runs a LabVIEW App (block 6 of Figure 1) to interface with the Waspmote card by I^2^C (see Section 2.4 for further details). This application requests data to the Waspmote every 200 ms. If the received packets are from the camera application (own position and orientation), the position is compared to the last broadcasted position, *i.e.*, to the state which generated the last event. If the difference is greater than a threshold, then the current position is sent to its neighbors by using the I^2^C interface with the Waspmote. By contrast, if the source is any robot in the neighborhood, the received data is then used to update the control law as explained later in Section 3.

The communication between the PC and the Waspmote is carried out by the Waspmote's Gateway (block 3 of Figure 1) connected to the PC through the USB port. The Waspmote's Gateway has drivers for Java but not for LabVIEW. For this reason, it is necessary to use an application developed in Java to send the data to each corresponding robot using the gateway. In this case, the JIL Server application [28] (block 11 of Figure 1) is used as interface between the camera application and the EJS Robots App (block 9 of Figure 1). This latter application sends the position and orientation to the robots by using XBee-Java-API (block 10 of Figure 1).

The data packet sent from the PC and between the robots has a size of 16 bytes. This is the maximum size allowed by NXT LEGO Mindstorms for the communications by I^2^C port [33].

**Figure 4.**
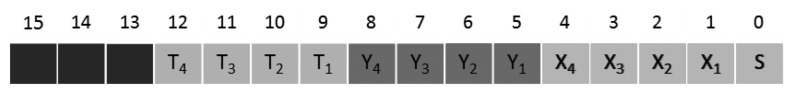
Data packet structure.

Figure 4 shows the packet structure, which is as follows:

- Byte 0 (S): The source where the packet comes from. The value is 0 for the robot 0, 1 for robot 1, and so on.
- Bytes 1… 4 (X_1_… X_4_): 4 bytes corresponding to the X coordinate of the robot position.
- Bytes 5… 8 (Y_1_… Y_4_): 4 bytes corresponding to the Y coordinate of the robot position.
- Bytes 9… 12 (T_1_…T_4_): 4 bytes corresponding to the robot orientation (θ).
- Bytes 13…15: Not used in this platform.

### 2.4. Using I^2^C in NXT LEGO

The LabVIEW toolkit for LEGO has buffers to write and read in each port, and three system call methods to access to these buffers. The write operation begins with the corresponding system call method, which constitutes the start of an asynchronous transaction between the NXT brick and the Waspmote. If the return value is zero, the method starts a communication transaction. After a write transaction is started it is necessary to use a system call function to check the status of the port. To ensure the success of the transmission, there is a system call function to check the status port.

The read operation of a device through I^2^C port is carried out in two stages. First, a write operation must be executed with the number of bytes that the slave has to send. If the count of bytes available in the read buffer is not zero, the read operation is executed and the data is obtained in the external buffer.

## 3. Event-Based Control and Communication in Multi-Agent Systems

### 3.1. Multi-Agent Systems and the Consensus Problem

The simplest model to represent the communication topology of a multi-agent system is a graph *G* = {*V,E*{, where the nodes *V* correspond to agents and the edges *E* represent communication links between nodes.

According to [3], a simple consensus algorithm to reach an agreement regarding the state of *N* single integrators of the form *x_i_*(*t*) ****=***u_i_*(*t*) can be expressed as an *nth* order linear system on a graph:

(1) $${\dot{x}}_{i}(t)=\sum_{j\in N_{i}}\left(x_{j}(t)-x_{i}(t)\right)$$

The dynamics of the group of agents can be written as:

(2) $$\dot{x}(t)=-Lx(t)$$

where *L* is the laplacian of the graph, and *N_i_* the set of neighbors of the node *i* [34].

Based on analytical tools from algebraic graph theory, it can be shown that if the graph is connected, then there is an unique equilibrium state for Equation (2) of the form *x_eq_*=α**1**, where $\alpha =\frac{1}{N}\sum_{i=1}^{N}x_{i}(0)$. and **1 =** (1 … 1)^T^.

Consensus algorithms can be extended to formation control if the formation is represented by vectors of relative positions of neighboring agents. In particular, let us denote by **r***_ij_* the desired inter-vehicle relative position vector. The following control law:

(3) $${\dot{x}}_{i}(t)=\sum_{j\in N_{i}}\left(x_{j}(t)-x_{i}(t)-{\text{r}}_{ij}\right)$$

yields the group to achieve the objective of the formation.

The consensus problem has been extending regarding the agents dynamics to higher order integrators [35,36], or linear systems [37].

### 3.2. Model of Non-Holonomic Mobile Robots

Single or double integrators do not describe properly the dynamics of most of commercial mobile robots, since these cannot move in any direction instantaneously. In robotics, holonomicity refers to the relationship between the controllable and total degrees of freedom of a given robot. If the controllable degrees of freedom are less than the total degrees of freedom the vehicle is non-holonomic. To avoid the non-holonomic constraint, Reference [38] defines the dynamics in terms of the front wheels coordinates (*x̄_i_*, *ȳ_i_*) as:

(4) $$\left(\begin{array}{c} {\dot{\bar{x}}}_{i}\\ \dot{\bar{y}}\\ {\dot{\theta }}_{i} \end{array}\right)=\left(\begin{array}{cc} \cos\theta_{i} & -d\sin\theta_{i}\\ \sin\theta_{i} & d\cos\theta_{i}\\ 0 & 1 \end{array}\right)\left(\begin{array}{c} v_{i}\\ \omega_{i} \end{array}\right)$$

where *x̄*_*i*_=*x*_*i*_+*d* cos*θ_i_, ȳ_i_*=*y_i_*+*d* sin *θ_i_*. The triple (*x*_*i*_, *y*_*i*_θ_*i*_) denotes the position and the orientation of the vehicle (see Figure 2b, *x_i_= Xcm, y_i_= Ycm* for each vehicle), and *vi* and *ω_i_* are the longitudinal and angular velocities, respectively. Let us consider as control inputs for each robot *u_i_*= (*v_i_*, *ω_i_*).

### 3.3. Time Schedule Control

In a distributed control approach, each agent collects information from its neighboring nodes and updates the control law according to some rules. In a networked system, collecting information means to transmit it through the communication channel. The information can be transmitted through the network in a periodic or an event-based fashion. The first case involves the traditional approach of sampling at pre-specified time instances, usually separated by a specific sampling period. However, the selected period must assure adequate system performance over a wide range of operating conditions. This can yield to a conservative choice and result in significant over-provisioning of the communication-network. Event-triggering represents one way of generating sporadic transmissions across network channels [39]. The communication is invoked only when something significant has occurred in the system.

The problem of multi-agent systems with event-based communications has been recently addressed [40,41]. Whereas the controller design of [41] is centralized, [40] presents a distributed approach, in which the agents are modeled by single and double integrators. The control law is based on the states that the agents broadcast to their neighbors through the network at event times. The average consensus is reached preserving convergence properties and the experimental results show a significant reduction in the number of transmissions respect to a periodic approach. Distributed event-based control has been also addressed in [42], where physical coupling may exist between the subsystems.

The control signals in each robot are computed based on the broadcasted information, which means that are not updated continuously, not even at each measurement of its own state received by the camera (see Section 2.2). The rule to determine when to broadcast is given by the *trigger function*. A trigger function determines the instants of time { $t_{k}^{i}$ } at which each agent broadcasts the state to its neighbors. In general, these broadcasting times are not equidistant as in periodic communications. Moreover, the proposed trigger functions depend on local information only.

If we denote the state of an agent *i* as *x_i_*(*t*) and *x̂_i_*(*t*) as the broadcasted state of agent *i* to its neighbors, which is a piecewise constant function, an event is triggered for an agent *i* when:

(5) $$f_{i}\left(x_{i}(t),{\hat{x}}_{i}(t),\underset{j\in N_{i}}{\cup }{\hat{x}}_{j}(t)\right)>0$$

is fulfilled, where *N_i_* is the neighborhood of agent *i*. The trigger functions are said to depend on local information because the decision of when to re-compute the control law depends on the last update values and the neighbors' states, and each agent does not have to monitor the states of its neighbors continuously in order to evaluate the triggering condition, in contrast to the trigger rules proposed in [10].

Specific trigger functions based on the error *e_i_*(*t*) ****=***x̂_i_*(*t*)*-x_i_*(*t*) are proposed. For example, static trigger functions have the form *f_i_*(*e_i_* (*t*)) **=** |*e_i_*(*t*)| *-c_0_*, where *c*_0_ is a constant value.

The control law *u_i_= (v_i_*, *ω_i_)* to reach the desired formation is computed as:

(6) $$\left(\begin{array}{c} v_{i}\\ \omega_{i} \end{array}\right)=-{\left(\begin{array}{cc} \cos\theta & -d\sin\theta_{i}\\ \sin\theta_{i} & d\cos\theta_{i} \end{array}\right)}^{-1}\left(\begin{array}{cc} L & 0\\ 0 & L \end{array}\right)\left(\begin{array}{c} \hat{\bar{x}}-{\bar{x}}_{r}\\ \hat{\bar{y}}-{\bar{y}}_{r} \end{array}\right)$$

where:

- $\hat{\bar{x}}$ and $\hat{\bar{y}}$ are the stack vectors of the broadcasted positions, *i.e.* ${\hat{\bar{x}}}^{T}=\left({\hat{\bar{x}}}_{1},\cdots ,{\hat{\bar{x}}}_{N}\right)$ and ${\hat{\bar{y}}}^{T}=\left({\hat{\bar{y}}}_{1},\ldots ,{\hat{\bar{y}}}_{N}\right)$.
- *x̄_r_* and *ȳ_r_* are the stack vectors of the desired positions of the agents with reference to the center of the group.

Combining Equations (4) and (6) it yields that:

(7) $$\begin{array}{c} \dot{\bar{x}}=-L(\hat{\bar{x}}-{\bar{x}}_{r})\\ \dot{\bar{y}}=-L(\hat{\bar{y}}-{\bar{y}}_{r}) \end{array}$$

### 3.4. Stability Anaalysis

Note that defining the control law given in Equation (6), two decoupled equations are obtained and they correspond to the equation of the event-triggered consensus problem of single integrators studied in [40], but extended to reach a formation. There it was proved that the consensus was reached, so in this case the agents achieve the desired formation translated into the XY plane by a vector which is the average of the initial positions (average consensus).

Hence, the stability results sum up in the following corollary to the Theorem 4 in [40], in which time-dependent trigger functions of the form:

(8) $$f_{i}(e_{i}(t))=\left|e_{i}(t)\right|-(c_{0}+c_{1}e^{-\alpha t})$$

are considered.

*Remark 1*. In trigger functions given in Equation (8) it is assumed that the initial conditions are *t*_0_ = 0. Otherwise, Equation (8) can be rewritten as:

(9) $$f_{i}(e_{i}(t))=\left|e_{i}(t)\right|-(c_{0}+c_{1}e^{-\alpha (t-t_{0})})$$

where *t*_0_ refers to the initial time of the experiment. Thus, the threshold decreases exponentially and takes the value *c*_0_ in the limit when time goes to infinity.

*Corollary 2* If trigger functions given in Equation (8) are defined for the errors in the x- and y-coordinates, such that:

(10) $$\begin{array}{c} e_{x,i}={\hat{\bar{x}}}_{i}-{\bar{x}}_{i}\\ e_{y,i}={\hat{\bar{y}}}_{i}-{\bar{y}}_{i}, \end{array}$$

the formation given by (*x̄_r_,ȳ_r_*) is reached with an error that depends on the value of parameter c_0_ of the trigger function, and the center of the formation is the average of the initial positions:

(11) $$\begin{array}{c} a_{x}=\frac{1}{N}\sum_{i=1}^{N}{\bar{x}}_{i}(0)\\ a_{y}=\frac{1}{N}\sum_{i=1}^{N}{\bar{y}}_{i}(0). \end{array}$$

*Proof*. Let us denote:

(12) $$\begin{array}{c} \delta_{x}=\bar{x}-a_{x}1-{\bar{x}}_{r}\\ \delta_{y}=\bar{y}-a_{y}1-{\bar{y}}_{r} \end{array}$$

the disagreement vectors in the *x*- and *y*-coordinates, respectively, where 1 = (1 … 1)^T^ is an eigenvector of the laplacian *L* [3]. Thus, it holds that ${\dot{\delta }}_{x}=\dot{\bar{x}},{\dot{\delta }}_{y}=\dot{\bar{y}}$, and it yields:

(13) $$\begin{array}{c} {\dot{\delta }}_{x}=-L(\delta_{x}+e_{x})\\ {\dot{\delta }}_{y}=-L(\delta_{y}+e_{y}), \end{array}$$

where *e_x_= (e_x,1_…e_x,N_)^T^* and *e_y_= (e_y1_…e_yN_)^T^*. Hence, applying the results of Theorem 4 in [40], it yields the disagreement vectors *δ_x_*,*δ_y_* converge to the following regions, respectively:

(14) $$\begin{array}{c} \left\|\delta_{x}\right\|\leq \frac{L}{\lambda_{2}(G)}\sqrt{Nc_{0}}\\ \left\|\delta_{y}\right\|\leq \frac{L}{\lambda_{2}(G)}\sqrt{Nc_{0}}, \end{array}$$

where λ_2_(*G*) is the algebraic connectivity of the graph *G* (second smallest eigenvalue of *L*).

*Remark 2*. Note that the convergence of the disagreement vectors *δ_x_, δ_y_* proves that the group of robots reaches the formation at (*a_x_***1*+****x̄_r_,a_y_***1*+****ȳ_r_*) with an error given by Equation (14).

Despite the fact that the existing theory that proves the robustness of the proposed control strategy and the simulation results that show a significant improvement as far as the number of transmissions between agents is concerned, there are neither experimental results on a real platform nor a simulation tool to perform a wide set of experiments of formation control of mobile robots. The following section describes the developed software and all its capabilities.

## 4. Simulation Tool

The many control and system configuration options needed to simulate a multi-agent system demand a graphical user interface (GUI) with a high degree of flexibility. The GUI is intended to make rapid prototyping and simulation of wireless autonomous agents which execute distributed control algorithms and perform event-based communications. Nevertheless, the GUI has been made keeping the interaction with the user relatively simple and intuitive in order to be used as a pedagogical tool for advanced engineering control courses. The simulation tool is available on line at http://lab.dia.uned.es/mass. See [43] for further details.

### 4.1. Description of the Simulator

**Figure 5.**
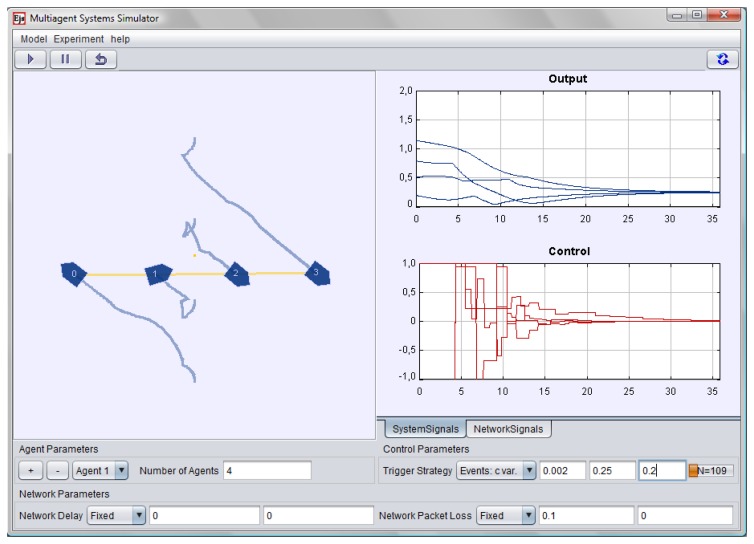
Illustrative example of the simulation tool. A multi-agent system with four agents is simulated. Note that the control actions are constant between two communication events.

The interface of the application, shown in Figure 5, has five main panels, a menu bar, and a small task bar. The two upper panels of the interface provide a quick view of the multi-agent system and a time plot of the outputs and control signals. The top left panel shows an animation of the complete multi-agent system. Each agent is numbered and shows a trace of its former positions. The lower left panel, named *Agent Parameters*, allows users to set the number of agents in the system, as well as to add and remove a particular agent.

The time plots on the top right panel, which are grouped in the *System Signals* tab, display the relative distance to the desired formation as well as the control actions of each agent. There are also plots grouped in the *Network Signals* tab, which provide information about the measured delay of the sent packets.

The lower panel, named *Network Parameters*, is devoted to setting up the network delay and also the network packet loss. Users can choose this panel to set fixed or random delay and loss probability functions. The lower right panel *Control Parameters* is used to specify the communication strategy which triggers the sending of packets from the agents in order to update the control actions. The interface is completed with a top task bar that provides buttons to start, pause, and reset the simulation.

The components of the interface described earlier provide the basic functionality required to operate the application. A menu bar provides some additional features such as the possibility to specify the dynamic model of the agents and to select a predefined multi-agent system configuration and the experiment to be performed. The user can also save a configuration, mark it as default or load a previous configuration.

### 4.2. A Practical Example

**Figure 6.**
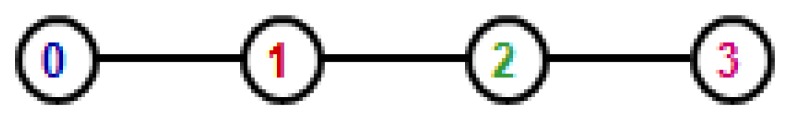
Communication graph describing one-dimensional network topology.

This section presents a usage example of the simulator described above. Different experiments are prefixed. We select one of them in the top menu, for example, *Experiment 2*, in which the communication topology for four vehicles is given by the graph in Figure 6, and the desired formation respect to the center of the group is:

(11) $$\begin{array}{cc} x_{r}=[\begin{array}{cccc} -0.7 & -0.25 & 0.2 & 0.65 \end{array}], & y_{r}=[\begin{array}{cccc} 0 & 0 & 0 & 0 \end{array}] \end{array}$$

The user can find in the help menu all the information about these experiments. Random initial conditions are generated, but we may be interested in comparing the performance of different control strategies (periodic and event-based, for example) for a prefixed initial coordinates. We can do that by right-clicking in the drawing panel and selecting the option “*Setting current state as default*”.

Figure 5 shows the simulation output when the trigger function is of the form *f_i_*(*e_i_*(*t*)) **=***|e_i_*(*t*)**|-**0.002 **+** 0.25*e^-^*^0.2^*^t^*. The user can change the parameters of *f_i_* while the simulation is running or switch to a periodic communication and check online the effect of these changes in the system.

## 5. Experimental Results

### 5.1. Comparison of Simulation and Experimental Results

**Figure 7.**
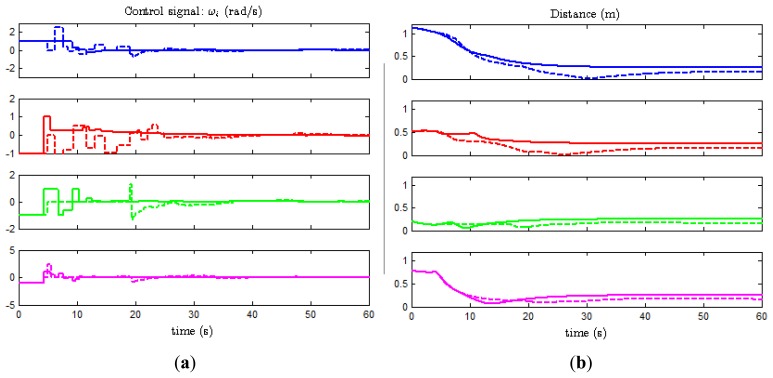
Comparative of simulation and experimental results: (**a**) Control signal, (**b**) Distance to formation.

Figure 7a,b shows the second component of the control signals (*ω_i_*) and the distances to the formation $d_{i}=\sqrt{{\left({\bar{x}}_{i}-{\bar{x}}_{i,r}\right)}^{2}+{\left({\bar{y}}_{i}-{\bar{y}}_{i,r}\right)}^{2}}$, respectively, for the four agents when the experiment described in Section 4.2 is performed over the model (solid lines) and the real system (dashed lines).

We observe that the major discrepancies are at the beginning of the experiment. Note that the control signals are constant piecewise functions and change their value only at event times, while Equation (6) reads that they change continuously with the orientation *θ_i_* even though $\hat{\bar{x}}-{\bar{x}}_{r}$ and $\hat{\bar{y}}-{\bar{y}}_{r}$ remain constant in the inter-event times. The reason for this is the constraint imposed by the hardware in the real system. Because one agent cannot monitor its state continuously (the camera sends measurements every 200 ms, which is the minimum period allowed by the Waspmote cards), and the slow variation of the angle, the computation required to implement the continuous control law given in Equation (6) is not worthy. Thus, the angle is considered to remain constant between events.

**Figure 8.**
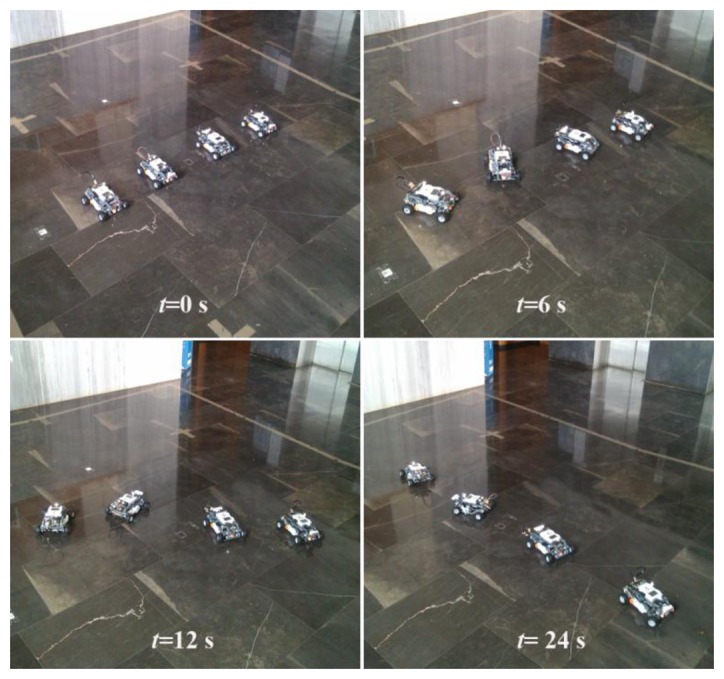
View of the real experiment at t = 0, 6, 12, and 24 s.

Moreover, the selected trigger functions decrease with time. This means that the threshold to detect an event is greater for small values of time, and fewer events are generated consequently. When the system approaches to the desired formation, the threshold decreases and makes the error arbitrary small. Note that *d_i_* takes the same value for all *i* = 1,…,4 and the final state only depends on the initial conditions, as predicted by the consensus problem theory (see Section 3). However, the final state for the real experiment differs slightly from the result given by the model. The reason for that might be sensing errors, dead-zone in the actuator and/or transmission delays. Finally, Figure 8 shows the view of the experiment at *t* = 0, 6, 12, and 24 s.

### 5.2. Event-Trigger Versus Time-Trigger

**Figure 9.**
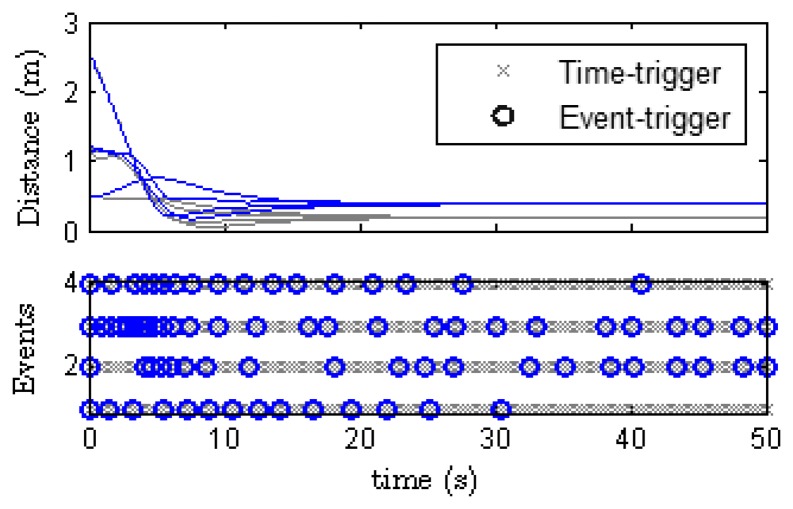
Comparison of the results obtained for event-trigger and time-trigger experiments.

Figure 9 compares the results obtained from event-trigger and time-trigger experiments. In the last case, each agent receives data from the camera every 200 ms, it processes the information, broadcasts its state to its neighbors, and computes the control law every two samples (400 ms). In the lower graph of Figure 9 the broadcasts in both cases are depicted for each agent (labeled from 1 to 4). For the periodic case, the number of packets sent through the network during the experiment should be *50s×*4*agents/*0.4 ****=** 500. However, for the event-based control this number is by far lower: 83 events in total.

In the upper graph of Figure 9 the distance to the formation is shown. Note that even though the desired formation and the initial conditions are the same, the final state (which depends on the average of the initial positions) is different. As remarked before, these differences come out because it is difficult in experimentation to exactly reproduce the same conditions.

For instance, network delays cannot be controlled, or sensing errors may occur more frequently in one case than in the other. However, observe that the time at which the formation is achieved (the stationary) is very similar for both time-trigger and event-trigger.

## 6. Conclusions

We have described an experimental environment for mobile robots. The hardware platform was developed using different components and software to overcome the limitations of the LEGO Mindstorms NXT robots. A distributed control approach has been used in formation control. Each robot communicates to its neighborhood in an event-triggering fashion for network saving purposes. A simulation tool with high degree of flexibility has been presented. An example has demonstrated its usability, and simulated and experimental results have been presented showing how the multi-agent system reaches the desired formation with few communications meanwhile the system performance is preserved. Future works will include the implementation of collision and obstacle avoidance control and formation control with specified paths. Exploring self-triggering techniques [44] to predict the next event occurrence in order to optimize the communication between the camera and the robots is also a future line of research.

## References

[1] Franchi L., Freda G.O., Vendittelli M. (2009). The sensor-based random graph method for cooperative robot exploration. IEEE ASME Trans. Mechatron.

[2] Magnenat S., Rétornaz P., Bonani M., Longchamp V., Mondada F. (2011). ASEBA: A modular architecture for event-based control of complex robots. IEEE ASME Trans. Mechatron.

[3] Olfati-Saber R., Fax J., Murray R. (2007). Consensus and cooperation in networked multi-agent systems. IEEE Proc..

[4] Lian F.-L., Moyne J., Tilbury D. (2002). Network design consideration for distributed control systems. IEEE Trans. Control Syst. Technol..

[5] Aström K., Bernhardsson B. Comparison of Riemann and Lebesgue Sampling for First Order Stochastic Systems.

[6] Tabuada P. (2007). Event-triggered real-time scheduling of stabilizing control tasks. IEEE Trans. Autom. Control.

[7] Rabi M., Johansson K.H., Johansson M. Optimal Stopping for Event-Triggered Sensing and Actuation.

[8] Dimarogonas D.V., Frazzoli E., Johansson K.H. (2012). Distributed event-triggered control for multi-agent systems. IEEE Trans. Autom. Control.

[9] Mazo M., Tabuada P. (2011). Decentralized event-triggered control over wireless sensor/actuator networks. IEEE Trans. Autom. Control.

[10] Wang X., Lemmon M.D. (2011). Event-triggering in distributed networked control systems. IEEE Trans. Autom. Control.

[11] Marchand N., Durand S., Guerrero-Castellanos J.F. (2013). A general formula for event-based stabilization of nonlinear systems. IEEE Trans. Autom. Control.

[12] Macdonald E.A. (2011). Multi-Robot Assignment Formation Control. M.Sc. Thesis.

[13] Huang J., Farritor S.M., Qadi A., Goddard S. (2006). Localization and follow-the-leader control of a heterogeneous group of mobile robots. IEEE ASME Trans. Mechatron.

[14] Mehrjerdi H., Saad M., Ghommam M.J. (2011). Hierarchical fuzzy cooperative control and path following for a team of mobile robots. IEEE ASME Trans. Mechatron.

[15] Cruz D., Mcclintock J., Perteet B., Orqueda O., Cao Y., Fierro R. (2007). A multivehicle platform for research in networked embedded systems. IEEE Control Syst..

[16] Durand S., Minet J., Guerrero J.F., Marchand N. ASYNCAR, a Radio-Controlled Vehicle for Asynchronous Experiments Implementation of an Event-Based Cruise Control.

[17] Casani M., Garulli A., Giannitrapani A., Vicino A. A LEGO Mindstorms Multi-Robot Setup in the Automatic Control Telelab.

[18] Maze M., Wan Y., Namuduri K., Varanasi M. (2012). A LEGO Mindstorms NXT-Based Test Bench for Cohesive Distributed Multi-Agent Exploratory Systems.

[19] Fu G., Corradi P., Menciassi A., Dario P. (2011). An integrated triangulation laser scanner for obstacle detection of miniature mobile robots in indoor environment. IEEE ASME Trans. Mechatron..

[20] Espinosa F., Santos C., Marrón-Romera M., Pizarro D., Valdés F., Dongil J. (2011). Odometry and laser scanner fusion based on a discrete extended kalman filter for robotic platooning guidance. Sensors.

[21] Chaos D. (2011). Control no lineal de vehículos subactuados marinos no-holonómicos. Ph.D. Thesis.

[22] Libelium Comunicaciones Distribuidas S.L. Waspmote Datasheet. http://www.libelium.com/downloads/documentation/waspmote_datasheet.pdf.

[23] LEGO Group LEGO Mindstorms NXT User Guide. http://mindstorms.lego.com/en-us/support/buildinginstructions/8547/8547%20User%20Guide%20English.aspx.

[24] HiTechnic IR RC Kart Building Instructions. http://www.hitechnic.com/file.php?f=503-HTRCKart.pdf.

[25] Robot Electronics Using the I2C_Bus. http://www.robot-electronics.co.uk/acatalog/I2C_Tutorial.html.

[26] Esquembre F. (2004). Easy Java Simulations: A software tool to create scientific simulations in Java. Comput. Phys. Commun..

[27] Raap, XBee-API A Java API for Digi XBee/XBee-Pro OEM RF Modules. http://code.google.com/p/xbee-api/.

[28] Vargas H., Sánchez J., Salzmann C., Esquembre F., Gillet D., Dormido S. (2009). Web-enabled remote scientific environments. Comput. Sci. Eng..

[29] Viguria A., Howard M. (2009). An Integrated approach for achieving multirobot task formations. IEEE ASME Trans. Mechatron..

[30] Digi International Inc. Product Manual v1.xEx—802.15.4 Protocol. http://ftp1.digi.com/support/documentation/90000982_K.pdf.

[31] Libelium Comunicaciones Distribuidas S.L. Waspmote Technical Guide. http://www.libelium.com/documentation/waspmote/waspmote-technical_guide_eng.pdf.

[32] Guascon D. (2010). Long Range Multiprotocol Wireless Sensor Networks.

[33] National Instruments Lab VIEW Toolkits for LEGO Mindstorms NXT Programming Guide. http://download.ni.com/evaluation/mindstorms/LabVIEW_for_NXT_Advanced_Programming_Guide.pdf.

[34] Godsil G.R. (2001). Algebraic Graph Theory.

[35] Ren W., Atkins E. (2007). Distributed multi-vehicle coordinated control via local information exchange. Int. J. Robust Nonlinear Control.

[36] Ren W., Moore K., Chen Y. High-Order Consensus Algorithms in Cooperative Vehicle Systems.

[37] Seo J.H., Shim H., Back J. (2009). Consensus of high-order linear systems using dynamic output feedback compensator: Low gain approach. Automatica.

[38] Lawton J.R.T., Beard R.W., Young B.J. (2003). A decentralized approach to formation maneuvers. IEEE Trans. Robot. Autom..

[39] Lemmon M. (2010). Event-Triggered Feedback in Control Estimation Optimization. Networked Control Systems.

[40] Seyboth G., Dimarogonas D., Johansson K. Control of Multi-Agent Systems via Event-Based Communication.

[41] Demir O., Lunze J. Cooperative Control of Multi-Agent Systems with Event-Based Communication.

[42] Guinaldo M., Dimarogonas D., Johansson K., Sanchez J., Dormido S. Distributed Event-Based Control for Interconnected Linear Systems.

[43] Guinaldo M., Farias G., Fabregas E., Sánchez J., Dormido-Canto S., Dormido S. (2012). An interactive simulator for networked mobile robots. IEEE Netw. Mag..

[44] Mazo M., Anta A., Tabuada P. (2010). An ISS self-triggered implementation for linear controllers. Automatica.

